# ped_draw: pedigree drawing with ease

**DOI:** 10.1186/s12859-020-03917-4

**Published:** 2020-12-09

**Authors:** Matt Velinder, Dillon Lee, Gabor Marth

**Affiliations:** Department of Human Genetics, University of Utah School of Medicine, Salt Lake City, UT USA

**Keywords:** Pedigree, Genetics, Mendelian, Family, Family studies, Generation, Inheritance, Drawing, Ped, Visualization

## Abstract

**Background:**

Pedigree files are ubiquitously used within bioinformatics and genetics studies to convey critical information about relatedness, sex and affected status of study samples. While the text based format of ped files is efficient for computational methods, it is not immediately intuitive to a bioinformatician or geneticist trying to understand family structures, many of which encode the affected status of individuals across multiple generations. The visualization of pedigrees into connected nodes with descriptive shapes and shading provides a far more interpretable format to recognize visual patterns and intuit family structures. Despite these advantages of a visual pedigree, it remains difficult to quickly and accurately visualize a pedigree given a pedigree text file.

**Results:**

Here we describe *ped_draw* a command line and web tool as a simple and easy solution to pedigree visualization. *Ped_draw* is capable of drawing complex multi-generational pedigrees and conforms to the accepted standards for depicting pedigrees visually. The command line tool can be used as a simple one liner command, utilizing graphviz to generate an image file. The web tool, https://peddraw.github.io, allows the user to either: paste a pedigree file, type to construct a pedigree file in the text box or upload a pedigree file. Users can save the generated image file in various formats.

**Conclusions:**

We believe *ped_draw* is a useful pedigree drawing tool that improves on current methods due to its ease of use and approachability. *Ped_draw* allows users with various levels of expertise to quickly and easily visualize pedigrees.

## Background

Pedigree files are text files that convey important information about the samples within a genetics or bioinformatics study [4]. This includes, at a minimum, for each sample: a kindred/family identifier, a sample identifier, a paternal identifier, a maternal identifier, a sex identifier (1 = male, 2 = female), and an affected status or phenotype identifier (1 = unaffected, 2 = affected) [3]. This text encoding is efficient for computational tools, but is difficult for a human user to visually parse and understand family and inheritance relationships. As such, bioinformaticians and geneticists typically rely on visual depictions of pedigrees where each individual is a node in a connected network, with: squares representing males, circles representing females, unshaded nodes representing unaffected samples and shaded nodes representing affected samples. Individuals in the same family generation are drawn at the same vertical height (ie, father and mother), while individuals in the subsequent generation (ie, children of the father and mother) are drawn below and connected to their respective parents. This accepted format allows for rapid and intuitive visualization of family structures and how affected status is segregating in the family (ie, de novo, dominant, recessive, X-linked modes of inheritance). This visual format becomes particularly useful in large, multi-generation pedigrees such as the Utah Centre d'Etude du Polymorphisme Humain (CEPH) dataset which comprises 603 individuals across 34 different large multi-generation families [2].

While a few pedigree drawing tools have been published previously, *ped_draw* addresses many of the deficiencies in current tools and provides numerous distinct advantages. *Pedigreejs* is perhaps the most fully featured and well powered pedigree drawing tool currently available [1]. It is written in Javascript and uses the d3 library to output visualizations of pedigree structures. It is an interactive and visual tool capable of being integrated into other web tools or used as a standalone on the app website. However, being written in Javascript d3 for the web, *pedigreejs* is not approachable to many users unfamiliar with Javascript or specific web programming visualization libraries such as d3. *Pedigreejs* requires the Javascript package manager npm (including grunt-cli) within a specific deployment and hosted environment. To deploy *pedigreejs*, an extensive knowledge of Javascript is required and numerous customization and visualization decisions need to be hard-coded by the user. *Kinship2* provides pedigree visualizations as a package written in the R programming language [5]. Similar to Javascript, R has a significant learning curve. As *Kinship2* is also merely an R package, it requires users to write R code (typically within an R integrated development environment such as RStudio, which needs to be installed separately) even in the most simple pedigree drawing usages. *Madeline 2.0* is another pedigree drawing tool, and boasts support across multiple operating systems [6]. However, *Madeline 2.0* has numerous dependencies in all installation scenarios, requiring Cygwin on Windows and apt, cmake, libssl-dev, libcurl-dev, libxml2-dev on Linux. On Linux in particular, these dependencies require sudo level user installation permissions that are often inaccessible on academic or shared computing resources.

Importantly, all of these published tools have significant installation dependencies, have substantial programming learning curves and lack a simple one command input–output approach. *Ped_draw* was designed to occupy a specific niche within this set of available software. All of *ped_draw*’s dependencies are installed by default in a typical Linux environment and a Docker image provides additional portability. *Ped_draw*’s simplified approach removes the need for customization and allows users to easily and rapidly visualize pedigrees directly from a provided ped file.

## Implementation

*Ped_draw* is solely implemented in Python and requires Python 2.7.15 or greater. Converting the dot output of *ped_draw* to an image is achieved using graphviz (not provided). Image outputs from graphviz can be visualized in any graphics viewing application.

## Results

The input for *ped_draw* is a ped file and the output is an image file (generated by graphviz). *Ped_draw* generates a dot file that can be passed directly as stdout to graphviz to generate an image file. *Ped_draw* is capable of visualizing complex ped files including multi-generation (3 or more) parent-pairs, multiple affected individuals, large numbers of children (8 or more per parent-pair) and multiple distinct families/kindreds in the same input ped file into a single image output.

*Ped_draw* can be used as a simple one-liner command as follows (where example.ped is an example pedigree file):

- Generate a dot formatted file (written to stdout):
  $ python ~ /bin/ped_draw/ped_to_dot.py example.ped
- Generate a png image of the pedigree by piping the *ped_draw* output to graphviz/dot:
  $ python ~ /bin/ped_draw/ped_to_dot.py example.ped | dot -T png -o example.png

**Fig. 1:**
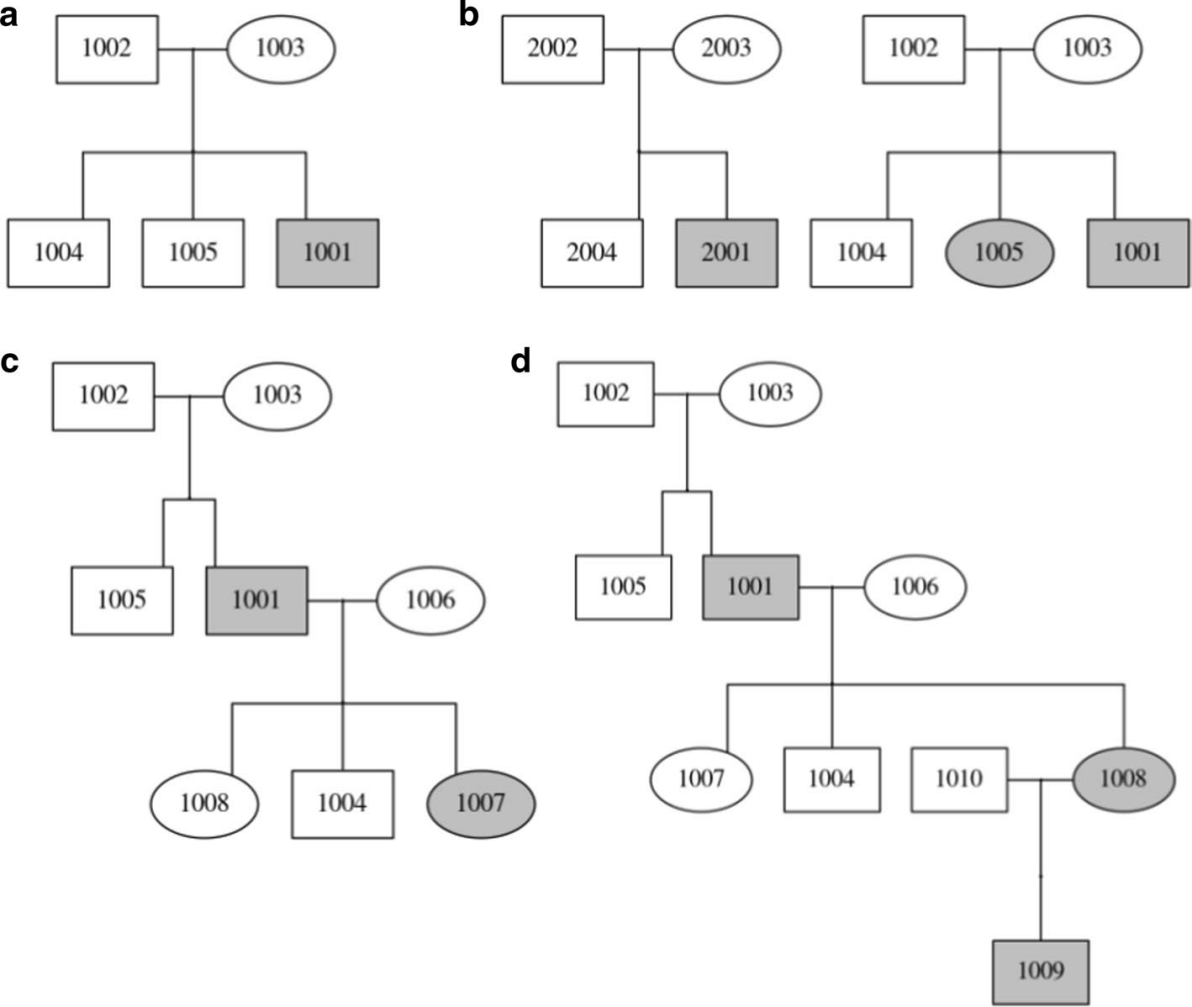
Example pedigree image outputs from *ped_draw*. **a** An example “quintet” single family pedigree. **b** An example pedigree of two distinct kindreds/families. **c** An example pedigree with three generations. **d** An example pedigree with four generations. The ped files used to generate these images are available in the GitHub repository under examples/

We demonstrate the ease and usage of *ped_draw* in multiple examples in Fig. 1. A relatively simple two generation pedigree with a single parent-pair and three children (one of which is affected) is drawn (Fig. 1a). Occasionally, ped files can contain multiple distinct families/kindreds, as specified by the first column of the file. This is interpreted by *ped_draw* and two distinct pedigrees are drawn (Fig. 1b). Pedigree files can also specify more than two generations. A three generation pedigree with multiple children in each generation and an unrelated father (node 1006) in generation 2 is drawn (Fig. 1c). An extended four generation family with multiple affected children across multiple generations is drawn (Fig. 1d).

In these examples and others, *ped_draw* draws the pedigree in the accepted “tree” representation, connecting all related nodes, assigning node shaped based on sex, labels nodes based on sample name and shades all affected nodes grey. This allows geneticists and bioinformaticians to readily visualize relatedness between samples and identify inheritance modes through multiple generations. Numerous other examples of pedigrees drawn by *ped_draw*, including pedigrees from the CEPH families, can be found on the Github repository under examples/.

## Conclusion

*Ped_draw* is a simple solution to the persistent bioinformatics and genetics challenge of visualizing pedigrees. *Ped_draw* is a robust and capable solution for drawing complex pedigrees within a simple Python and graphviz command. Both Python and graphviz are widely used, accessible, long-maintained and portable programming solutions. We anticipate *ped_draw* will be useful for both experienced bioinformaticians (using the command line tool) and users with little or no computational expertise (using the web tool).

## Availability and requirements

- Project name: ped_draw.
- Project home page: https://github.com/mvelinder/ped_draw and http://peddraw.github.io
- Operating system(s): UNIX (command line tool); platform-independent (web tool)
- Programming languages: Python, Javascript, HTML
- Other requirements: Python 2.7.15 or greater (command line tool), conversion of ped_draw output dot files to pngs or other image types requires graphviz, visualization of graphical outputs can be done by any number of graphics viewing applications; Chrome 80.0.3987.149 or greater (web tool)
- License: MIT-license
- Any restrictions to use by non-academics: MIT-license

## Data Availability

https://github.com/mvelinder/ped_draw and http://peddraw.github.io

## Abbreviation

CEPH: Centre d'Etude du Polymorphisme Humain
